# Induced Wood-Inorganic Composites in Standing Trees via Slow-Release Drip

**DOI:** 10.3390/polym14153103

**Published:** 2022-07-30

**Authors:** Jiangtao Shi, Haizhe Zhang, Yuhan Liu, Chongyang Xia, Yaoli Zhang

**Affiliations:** 1 Co-Innovation Center of Efficient Processing and Utilization of Forest Resources, Nanjing Forestry University, Nanjing 210037, China; zhangyaoli@126.com; 2 Department of Wood Science and Engineering, College of Materials Science and Engineering, Nanjing Forestry University, Nanjing 210037, China; zhz1459517843@163.com (H.Z.); liuyh@njfu.edu.cn (Y.L.); chongyang97@126.com (C.X.)

**Keywords:** living tree modification, TEOS, wood anatomy, cell wall formation, SiO_2_

## Abstract

It is a novel idea to fabricate wood-inorganic composites by utilizing the transpiration of bionic trees to realize the self-assembly of inorganic precursors in wood formation. We selected a 10-year-old poplar and diffused the solvent or sol containing SiO_2_ precursor into the xylem via the slow-release drip method. In combination with the moisture in xylem, reactions such as hydrolysis, polycondensation and self-assembly were induced in order to form wood inorganic composites. It was found, through microscopic observation, that such inorganic substances were yellowish brown and widely existed in vessels, wood fibers and ray cells. For the new grown wood, the fiber–tissue ratio and cell wall thickness underwent an increase, while the vessel diameter and tissue ratio experienced a decline. Moreover, such change was related to the concentration of precursors. EDS analysis proved that the elemental composition of sediments in wood cells was C, O, Si, K and Ca. XPS confirmed that the newly formed wood contained silicon oxide, illustrating that the standing tree slow-release drip technology could induce wood to fabricate inorganic composites.

## 1. Introduction

Plantation wood is an important source to alleviate the contradiction between the supply and demand of wood resources, but compared with natural forest wood, it has shortcomings such as loose property, low density, poor dimensional stability, perishableness [1], etc., which limit its application in wooden products. Usually, the properties of fast-growing wood can be improved or enhanced through various techniques such as resin impregnation, densification, heat treatment, acetylation, etc. However, these methods are all aimed at harvested wood, and their processing methods and chemical agents have problems such as potential environmental pollution, energy consumption, etc., which runs counter to the excellent characteristics of wood—a sustainable, green carbon sequestration material. Therefore, exploring green and efficient processing methods and modification systems has always been the priority of scientific researchers in this field [2,3].

Some wood is rich in inorganic minerals composed of calcium and silicon, the presence of which increases the density and hardness of wood [4]. Affected by factors such as tree species, minerals’ location within the tree trunk and so on, the distribution and content of these minerals in wood vary [5,6,7]. These differences are actually formed during the wood formation process, the mechanism for which is that the inorganic material, as reinforcement, is dispersed into the wood matrix so as to obtain a wood inorganic composite affecting its properties. However, the deposition of an inorganic substance is mainly attributed to the fact that cations and anions from the soil, which are transported into wood tissue through the cell pores of root and xylem, achieve biomineralization by self-organization in the parenchymal cell of wood. Just like seeds of inorganic substances, these cations and anions will experience four procedures of preorganization of the organic matrix, interface molecular recognition, growth modulation and epitaxial growth under the natural conditions of normal temperature and pressure to obtain organic/inorganic composites with excellent properties [8,9]. Revealing the biological formation process of these composite offers a new idea for artificially synthesized wood surface functional materials under in vitro conditions [10,11].

Biomineralization is a “living” life system that can be adopted as a new model for standing tree modification. Recently, Liu [12] delivered alkaline copper quaternary (ACQ) into plantation poplar wood by perforating in a tree stem and investigated the effect factors on the flow rate of the ACQ preservative. Shi et al. [13] injected tetrabutylorthotitanate (TBOT)/absolute ethanol into poplar stem and found that a roughly spherical and uniform dispersion of TiO_2_ particles formed in the wood vessel. Similarly, Furuno et al. [14] dripped a mixture of formaldehyde and urea into xylem to improve the wood mechanical properties of *Populus alba* var. In summary, research on the fabrication of woody composites in standing trees inspired by the biomineralization of the tree itself is still limited. In this study, poplars were taken as research objects, and the slow-release drip method was adopted to inject tetraethyl orthosilicate ethanol solution into the xylem of the poplars. By focusing on anatomical changes in the newly grown wood, this study aims to determine the chemical composition contained in wood tissue by X-ray photoelectron spectroscopy (XPS) and energy spectroscopy (EDS). It is expected to provide experimental grounds for the manufacture of wood-inorganic composites through the technology of the biomimetic mineralization of living wood.

## 2. Materials and Methods

### 2.1. Materials and Reagents

Ten-year-old Italy-Poplars growing in the Xiashu Internship Forest Farm (32°6′ N, 119°12′ E) of Nanjing Forestry University were selected for the study. Tetraethyl orthosilicate and xylene were purchased from Shanghai Aladdin Biochemical Technology Co., Ltd., Shanghai, China; Ethanol (Ethanol, mass fraction ≥ 99.7%) was purchased from Nanjing Chemical Reagent Co., Ltd., Nanjing, China; and neutral balsam was purchased from Shanghai Yiyang Instrument Co., Ltd., Shanghai, China, Deionized water was prepared by the deionized pure water machine numbered Master-Q in a laboratory.

**Figure 1:**
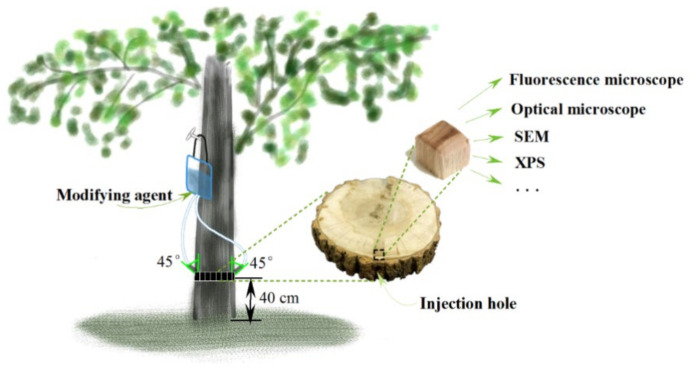
Schematic diagram of poplar living tree modification.

A total of 1 L of ethyl orthosilicate/ethanol solution was prepared one day before the impregnation treatment, with concentrations of 1 mmol/L, 10 mmol/L and 100 mmol/L, respectively. In addition, 10 mmol of tetraethyl orthosilicate and 10 mmol of absolute ethanol were added to 1 L of deionized water and magnetically stirred at room temperature for 5 min to prepare the SiO_2_ colloid. The prepared solution system was transferred into sap bags, and slow-release drip was conducted, as shown in Figure 1.

### 2.2. Slow-Release Drip in Trees

Poplars were divided into five groups: the untreated group (C); the 1 mmol/L ethyl silicate ethanol solution treatment group (T1); the 10 mmol/L ethyl silicate ethanol solution treatment group (T2); the 100 mmol/L ethyl silicate ethanol solution treatment group (T3); and the SiO_2_ colloid treatment group (S). Each group worked on three trees repeatedly. At a height of 40 cm above the ground, two holes were drilled symmetrically and downward with a depth of approx. 3.5 cm and an axial tilt of 45°. The sap bags were hung on the sunny side and then fixed (as shown in Figure 1). The flow rate was set at 0.01 mL/s. The slow-release drip started in July 2019 and ended in October of the same year, during which a new treatment solution was arranged to replace the old one every 7 days. Taking the injection hole as the center, a 10 cm thick disc was cut out and brought back to the laboratory for preservation. Similarly, a disc was cut out at the same height for the untreated group.

### 2.3. Optical Observation

The xylem (junction of the dark area and light area) was selected near the injection hole and the bark in the disc for the slow-release treatment group, and the xylem that the disc corresponded to in the same year was selected for the untreated group, with a size of 5 mm × 5 mm × 5 mm (longitudinal × radial × tangential). A cross-section of 15μm was cut by a slide slicer. Gradient dehydration was conducted with 30%, 50%, 70%, 90% and 100% aqueous ethanol, and it was soaked with xylene:anhydrous ethanol with a volume ratio of 1:1 for 1 min. It was cleared with xylene for 15 s and sealed with neutral balsam. It was observed and photographed under an ordinary optical microscope and fluorescence microscope, respectively. Cells such as vessels, wood rays and wood fibers were selected after image segmentation of 10× optical photos by Dragonfly 4.0, and the vessels and fiber tissue proportion were calculated. The double wall thickness of the fiber was measured by Oplenic software.

### 2.4. Microfibril Angle Determination

The MFA was determined by the iodine crystallization method. The wood radial section was immersed into the mixed solution of 10% nitric acid and 10% chromic acid with a volume ratio of 1:1 for about 15 min and rinsed with water until neutral. The section was placed on a glass slide, 2–3 drops of 6% potassium iodide solution were dripped and, finally, 65% nitric acid was added. It was photographed and measured by an optical microscope. A total of 50 sets of data were tested in each group, and the mean value was calculated.

### 2.5. Characterization

The cross section was flattened with a sliding slicer (consistent with the section position) and lyophilized. It was photographed and observed with the environmental scanning electron microscope FEI Quanta200 after metal spraying. In the meantime, the chemical composition of the micro area was analyzed by EDS. A 100 μm wood slice that had the same position as the slice was prepared, lyophilized and tested by a Bruker VERTEX 80/80 v FTIR spectrometer. The spectrometer was adjusted to mid-infrared reflection mode (ATR). The spectral resolution was set to 4 cm^−1^. It was scanned 16 times. Then, 4 mm × 4 mm × 1 mm (radial direction × tangential direction × longitudinal direction) wood chips were made with the remaining wood after slicing, and they were dried by lyophilizer. A wide-scan was performed on each slice with an AXIS UltraDLD photoelectron spectrometer equipped with an Al/Ag monochromatic dual-anode X-ray source at 600 W. A narrow scan was conducted after confirming the presence of C, Si, O, K and Ca, and the energy spectrum data were recorded. All the data were analyzed by Excel 2016 and plotted by Origin 2016.

### 2.6. Mechanical Strength Test

A tangential section was cut out of the xylem near the injection hole and the bark (the junction between the dark- and light-colored areas) with a slide slicer from the disc of the slow-release treatment group. The thickness, width and length for the tangential section were 100 μm, 5 mm and 5 cm, respectively. For each treatment group, ten samples were tested on a tensile mechanics tester with a spacing of 20 mm and a tensile speed of 5 mm/min. After that, the elastic modulus and tensile strength of each sample were automatically calculated by the machine, and then the average value was calculated.

## 3. Results

### 3.1. Macroscopic Observations of the Cross Section

**Figure 2:**
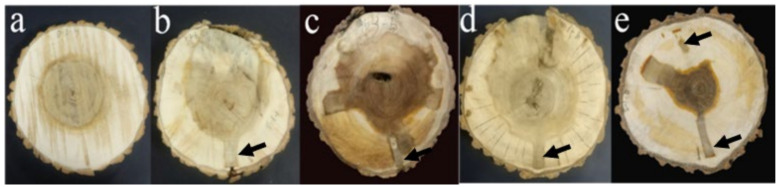
Macroscopic pictures of the cross section of *Populus euramevicana* discs (**a**) for the unmodified poplar disc, (**b**–**d**) for the 1, 10, 100 mmol/L TEOS injection group and (**e**) for the SiO_2_ colloid modified poplar disc. The black arrow represents the injection pore.

Figure 2 shows the difference in the surface color of the air-dried wood disc after slow-release dripping into the tree stem. Untreated poplar wood presented a yellowish white color in sapwood and a yellowish brown color in heartwood. There was an obvious boundary between the sapwood and heartwood which was shaped like a regular circle, and the proportion was about 26.3%, as shown in Figure 2a. On the contrary, both the color and shape of the heartwood-like wood were changed in the slow-release dripped wood. The color of the 1 mmol/L TEOS-treated wood group was similar to that of the reference group, but the yellowish brown area in the heartwood-like wood increased by 45.4%. Since the liquid flow was transmitted in the direction of the height of the trunk, the wood color changed to yellowish brown at the location of the injection hole (as shown in Figure 2b). The heartwood-like area of the 10 mmol/LTEOS-treated group presented an irregular shape, taking up an area of 42.8% or so, and, in multiple places of the sapwood, dark brown strips were formed, as shown in Figure 2c. This might be correlated with the decay cavity at the position of the heartwood. No significant differences were found in terms of the wood color of the 100 mmol/L TEOS-treated group and that of the reference group. However, occupying an area of about 47.2%, the irregular shape of the heartwood-like area did not expand, as shown in Figure 2d. 

In the SiO_2_ colloid modification group, the heartwood had a color of dark brown, and there were obvious traces of impregnating liquid remains and an injection pore position in the distribution area. However, compared with those three groups treated with TEOS, the heartwood-like area in this group took up a smaller proportion of 16.4%, as shown in Figure 1e. In the living tree, the transmission of slow-release dripped liquid in the axial and lateral directions was mainly caused by tree transpiration and permeability, respectively. After the standing tree was drilled and impregnated, the liquid was distributed in the trunk in an irregular way. This was basically correlated with the molecular weight, concentration and growth environment of the impregnating liquid [12,15], among which the concentration had the biggest influence on the lateral diffusion of the liquid. A previous work stated that liquid concentration was negatively correlated with penetration depth in lateral transportation [16]. On the contrary, our result proved an increase in penetration with the TEOS concentration in the lateral direction. In the xylem, the lateral transport of a substance basically depends on the laterally arranged ray tissue and the pits between the cells. In poplar, however, both the single row of rays and the size of the cell wall pits exerted a certain limit on the lateral diffusion of the liquid. Therefore, it was hard for the prepared sol-gel system to achieve a rapid spread laterally in the xylem; instead, a relatively small heartwood-like area was produced. Meanwhile, the reason why the wood experienced color changes might be the fact that new chemicals were generated in the wood out of the induction of impregnation. Examples were given to show that the affected wood resulting from different forms of damage can be chemically different from normal heartwood [17].

### 3.2. Anatomical Characteristic of Newly Formed Wood

**Figure 3:**
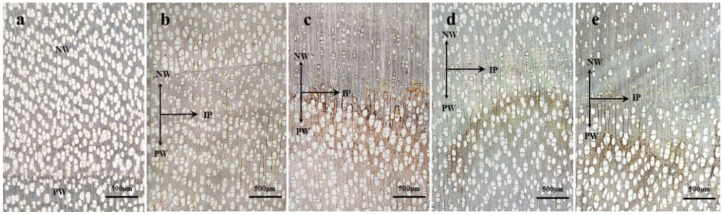
Cross section of poplar (4×), (**a**) for the unmodified treatment group, (**b**–**d**) for the 110,100 mmol/L TEOS modification treatment group, and (**e**) for the SiO_2_ colloidal modification treatment group. PW for previously grown wood, IP for injection point, NW for newly grown wood.

Poplar is a diffuse-porous wood. Its solitary and radial multiple pores were arranged in a scattered pattern, as shown in Figure 3a. After slow-release impregnation, the dimension and arrangement of the cells for newly grown wood went through a significant change. In the 1 mmol/L TEOS modification treatment group, both the number and diameter of pores had a slight decrease, but false growth rings were formed. Later, the wood cells tended to be normal, as shown in Figure 3b. In the 10 mmol/L TEOS treatment group, the number and diameter of pores underwent a similar decrease. A yellowish brown deposit appeared in the previously grown wood, spreading over in fiber and ray cells, as shown in Figure 3c. As for the 100 mmol/L TEOS treatment group, the wood pores presented relatively small diameters at the initial stage of treatment but gradually returned to normal at a later stage. Similarly, yellowish-brown deposits appeared in the cells of the grown wood, as shown in Figure 3d. In the SiO_2_ colloidal modification treatment group, there was also an obvious demarcation between the newly grown wood and the previously grown wood, with the former going through a decrease in the number and diameter of pores and the latter having yellowish brown deposits in the wood cells, as shown in Figure 3e.

The data for the newly grown wood of poplar, including the cell–tissue ratio, cell wall thickness, etc., is shown in Table 1. It was found that, after treatment, the proportion of vessel tissue in the newly grown wood was less than that of the untreated group. In a single plant, the newly grown tissue had a smaller proportion of vessel tissue compared with that of the injection point and previously grown wood. The newly grown wood in the 1 mmol/L TEOS treatment group went through the greatest decrease in the vessel tissue proportion, which was 15.08% and 19.14% lower than that of the untreated group and that of PW in the T1 group respectively. The 100 mmol/L TEOS treatment group experienced the smallest decline (3.41%) compared with the untreated group, and there was a 7.84% decrease within the T3 group compared with the PW. After the sol treatment, the vessel tissue proportion in the NW had a decrease of 19.24% and 14.36% compared with that of the untreated group and that of PW, respectively. In contrast, the fiber proportion in the NW underwent a certain degree of increase compared with that of the untreated group. Among the treated groups, the fiber–tissue ratio in the T2 group had the highest degree of increase, which was 17.77% higher than that of the untreated group and 17.20% higher than that of PW in the T2 group. As for the 100 mmol/L TEOS treatment group, only a slight change occurred in the fiber proportion—to be specific, a 2.51% increase over that of the untreated group and 11.56% higher than that of the PW within the T3 group. After the sol-gel treatment, the wood fiber tissue proportion of the NW was 13.75% and 11.56% higher than that of the untreated group and that of PW, respectively. After treatment, the thicknesses of the double cell walls were larger than those of the PW and those at the time of injection. The 1 mmol/L TEOS treatment group had the most significant differences with regard to its three locations. Compared with that of PW, the fiber double wall thickness for the NW increased by 3.29 μm, which was 0.67 μm higher than that of the untreated group. After the sol-gel treatment, the average fiber double wall thickness was 7.58 μm, which increased by 1.28 μm and 2.16 μm, respectively, compared with that of the untreated group and that of the PW. Since the density and strength of the wood were inversely correlated with the vessel tissue proportion and positively proportional with the fiber–tissue ratio and fiber wall thickness, it was speculated that the density and strength of the treated wood would increase, and those of the 10 mmol/L TEOS treatment group and SiO_2_ colloidal injection group had a better degree of increase than the other two groups, i.e., the 10 mmol/L and 100 mmol/L TEOS modification treatment groups.

**Figure 4:**
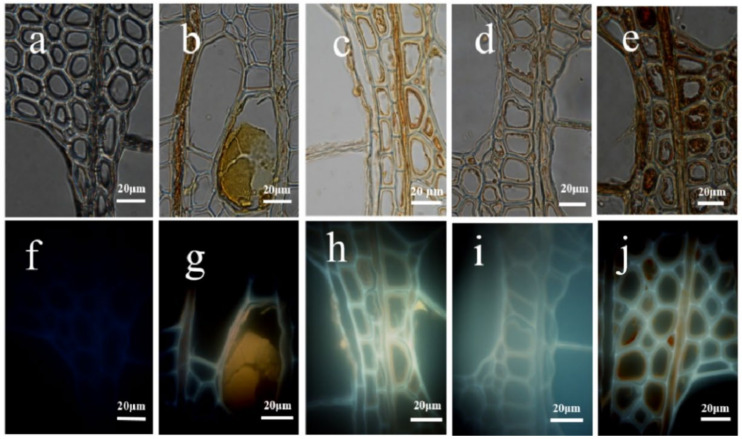
Optical and fluorescence photos of the poplar cross section under different treatment conditions. (**a**–**e**) are optical images, and (**f**,**j**) are fluorescent images**;** (**a,f**) for the unmodified treatment group, (**b**,**g**,**c**,**h**,**d**,**i**) for the 110,100 mmol/L TEOS modification treatment group and (**e**,**j**) for the SiO_2_ colloidal modification treatment group.

Under the optical microscope with the higher magnification, no obvious inclusions were observed in the vessel and fiber cells of the untreated poplar (as shown in Figure 4a). After the slow-release treatment, yellowish-brown inclusions appeared in the vessels, rays and wood fibers of the poplar, the amount of which was related to the concentration of the precursors. The inclusions, after the 1 mmol/L TEOS treatment, were mainly distributed in some vessels and rays and agglomerated in the vessel lumen, as shown in Figure 4b. If the 10 mmol/L and 100 mmol/L TEOS treatment was conducted, the inclusions would be distributed on the inner walls of the vessels, fibers and rays, appearing in a granular shape, as shown in Figure 4c,d. If the SiO_2_ colloidal modification treatment was carried out, there would be more abundant inclusions in the vessels, fibers and rays, presenting a similar granular shape, as shown in Figure 4e. Under the fluorescence microscope, the cell wall of poplar was found to be blue. This was mainly related to the spontaneous fluorescence of the unsaturated groups, such as the lignin and phenols in the cell wall at different wavelengths [18]. However, the fluorescence intensity of the cell wall was significantly different after treatment, with a greater one for the pale blue light in the intercellular layer and cell corner and a weakening one in the inner cell wall. The inorganic substances deposited in the cell lumen were yellowish brown and even reddish brown after being treated with the SiO_2_ colloid. The fluorescence phenomenon of the cell wall was not only related to fluorescence intensity but also correlated with the fluorescence concentration, composition and presence of the interacting surrounding molecules [19,20]. Therefore, it was inferred that the newly generated sediment after treatment directly affected the fluorescence properties of the cell wall [21].

### 3.3. Microscopic (SEM) Analysis and Energy Spectroscopy (EDS) Analysis

**Figure 5:**
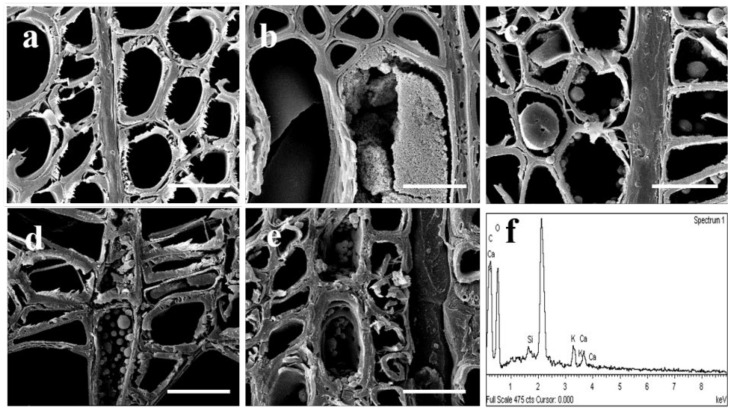
SEM images of the poplar cross section under different treatment conditions, (**a**) for the unmodified treatment group, (**b**–**d**) for the 110,100 mmol/L TEOS modification treatment group and (**e**) for the SiO_2_ colloidal modification treatment group; (**f**) for vessel the EDS spectrum, bar 20 μm.

As shown in Figure 5, the SEM results also demonstrated that, after the slow-release impregnation treatment, sediments in different shapes were formed in wood vessels, fibers and ray cells. Moreover, the distribution and aggregation patterns of the sediments were related to the concentration and type of precursors. The inclusions, after the 1 mmol/L TEOS treatment, were in blocky agglomerates in some vessels, as shown in Figure 5b. After the treatment with 10 mmol/L and 100 mmol/L TEOS, separate spherical sediments with diameters ranging from hundreds of nanometers to several microns were attached to the inner walls of the vessels, fibers and rays. There existed columnar or blocky sediments in some cells, as shown in Figure 5c,d. After the SiO_2_ colloidal modification treatment, the sediments appeared as having a spherical shape and tended to aggregate with each other, as shown in Figure 5e. 

It was found, through observation, that the spherical sediments mostly coincided with the pores on the cell wall, indicating that the precursors moved between wood cells. The more sediments in the ray also indicated that it was easier for the precursors to be deposited in the lateral transport process of the wood formation. A possible reason for such phenomenon could be the morphological difference of the sediments in the vessels and pores. As the main channels driven by transpiration, the vessels were inclined to accumulate in a large area, whereas the fiber cells, being blind-ended, could only be transmitted through the pores, and, thus, it was easy for them to deposit at the pits. Such deposition rule of the inorganic substances in wood cells conforms to the findings of Liu et al. [12]. The 1 mmol/L TEOS treatment only had substantial sediments in some vessels, and it probably occurred because the solution with a lower concentration was transported to other tissues of the trees with the trunk sap flow. When the 10 mmol/L and 100 mmol/L TEOS treatment was conducted, the precursors with an excessive concentration would be transported between cells via the pores with the liquid flow, producing hydrolysis with the water in the liquid flow. As a result, a spherical structure would be formed out of the influence of the water content and microscopic space. SiO_2_ sol had already appeared as spherical particles at the time of injection; therefore, it tended to deposit and agglomerate during the transportation between cells. The above discoveries suggested that the structure and morphology of the inorganic substances might be influenced by the type and concentration of the precursors, as well as by the tree sap flow.

According to EDS analysis, these sediments had similar chemical compositions, primarily for C, Si, K, Ca and O. The slow-release drip technique (exogenous substance invasion, trunk drilling) adopted by this study, as shown in Figure 5f, serves as an abiotic stress for the tree itself, disrupting the metabolic balance of the substances in the wood-forming tissues. The abiotic defense mechanism of the trees was activated, and the inorganic crystals existing in plants was involved in ion balance, adversity defense, strength support, etc. [22]. Ca^2+^ connected the external stimuli and physiological responses within a certain range to accumulate within the cells, reducing the Ca concentration in the apoplasts adjacent to the cells [23]. The SiO_2_ deposition sites on the cell wall in the silica-loving plants contained high contents of K and Cl. Therefore, in this experiment, inorganic substances such as Si, K and Ca were deposited in the wood cells.

### 3.4. Infrared Spectroscopy (FTIR) Analysis

**Figure 6:**
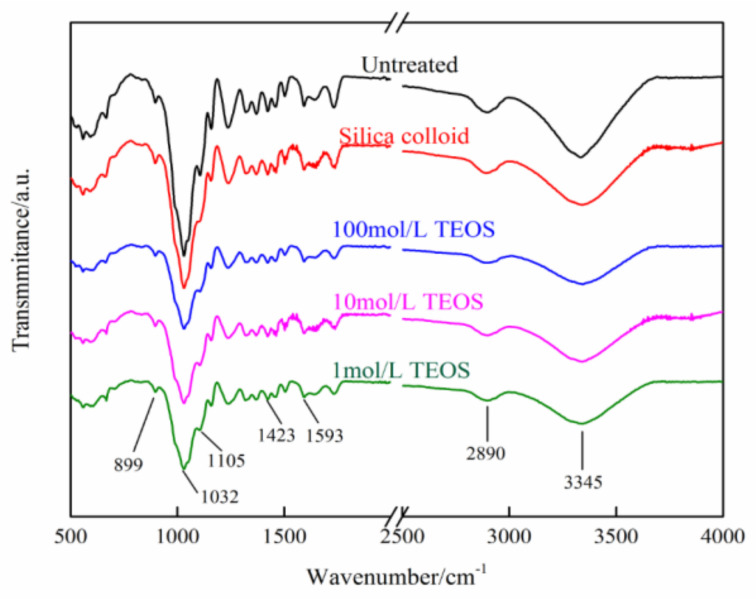
FTIR images of the poplar cross section under different treatment conditions.

The FTIR in Figure 6 indicated that, when the slow-release drip was conducted on the standing tree, it would form the wood/inorganic composites. The absorption peaks of the untreated samples were mainly caused by the vibration of functional groups on cellulose, hemicellulose and lignin in wood. The bands at 3345 cm^−1^ and 2890 cm^−1^ were assigned to O-H stretching vibration and C-H stretching vibration, respectively. Both the O-H and C-H groups were present in cellulose, hemicellulose and lignin. The band near 1730 cm^−1^ was assigned to the C=O stretch vibration of the acetyl or carboxylic acid groups, which were mainly associated with hemicellulose in wood tissue. The bands at 1593 and 1510 cm^−1^ were known as the characteristic vibration of the benzene ring skeleton from lignin. It was not easy for other bands at the “finger region” (1400–500 cm^−1^) to receive a qualitative analysis because these chemical groups might be associated with whole polymers in the wood cell wall. Compared with wood tissue in the untreated tree, the infrared spectra of the new wood inorganic composites had absorption peaks at similar positions, but the intensity for all the absorption peaks declined, indicating that the inorganic substances generated after treatment did not chemically react with the cell wall of the wood to form new chemical functional groups. With the TEOS concentration increasing, the intensity of the main absorption peaks underwent a slight decrease.

### 3.5. Poplar XPS Results

**Figure 7:**
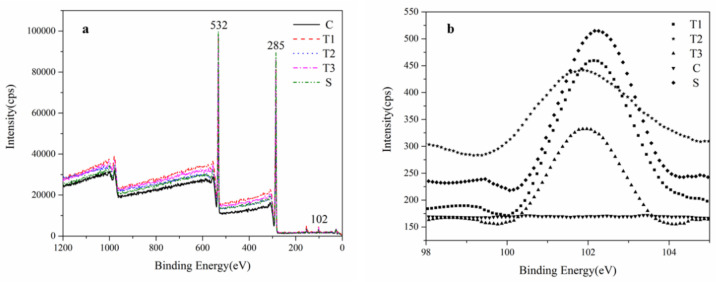
XPS wide scanning spectrum (**a**) and the scanning spectrum (101–102 eV) of silicon (**b**) in poplar. T1, T2, T3 for newly formed wood after the 1,10,100 mmol/L TEOS treatment, C for the newly formed wood of the untreated tree, S for the newly formed wood of the SiO_2_ colloidal injected tree.

Figure 7a shows the XPS wide spectra of wood under different treatment conditions. As shown in this figure, the absorption peaks near the binding energies of 284–290 eV, 531–534 eV and 102 eV are those for C, O and Si elements respectively. By magnifying the characteristic peaks for the silicon element at 102 ev (Figure 7b), it could be found that all the wood treated with slow-release drip generated obvious characteristic peaks of Si, indicating that silicon oxides had indeed been produced. Due to the presence of water in wood tissue, ethyl orthosilicate underwent hydrolysis-polycondensation with it, the reaction process for which is shown in the following four equations. Equation (5) embodied the general reaction of hydrolysis-polycondensation, from which it was learned that the whole reaction consumed two moles of water. Due to the uneven distribution of water in the trees and the presence of a variety of inorganic ions, the final content of SiO_2_ varied. Hence, as was observed in the XPS spectra, there was no clear relationship between the concentration of ethyl orthosilicate and the intensity of the characteristic peaks of Si.

(1) $$\mathrm{Si}{\left({\mathrm{OC}}_{2}H_{5}\right)}_{4}+H_{2}O\;\to \;\mathrm{Si}{\left(\mathrm{OH}\right)}_{4}+C_{2}H_{5}\mathrm{OH}\;$$

(2) $$\mathrm{Si}{\left(\mathrm{OH}\right)}_{4}\;+\;\mathrm{Si}{\left(\mathrm{OH}\right)}_{4}\;\;\;\to {\left(OH\right)}_{3}SiOSi{\left(OH\right)}_{3}+H_{2}O$$

(3) $$\mathrm{Si}{\left(\mathrm{OH}\right)}_{4}\;+{\left(OH\right)}_{2}\mathrm{Si}{\left(OC_{2}H_{5}\right)}_{2}\;\;\;\to {\left(OH\right)}_{3}SiOSi{\left(OH\right)}_{3}+C_{2}H_{5}OH$$

(4) $$n\left(\mathrm{Si}-O-\mathrm{Si}\right)\to {\left(\mathrm{Si}-O-\mathrm{Si}\right)}_{n}$$

(5) $$\mathrm{Si}{\left({\mathrm{OC}}_{2}H_{5}\right)}_{4}+2H_{2}O={\mathrm{SiO}}_{2}+4C_{2}H_{5}\mathrm{OH}\;$$

### 3.6. Analysis of the Microfibril Angle Change

As shown in Table 2, compared with the reference group, the microfibril angles of the wood under four types of treatment conditions presented varying degrees of increases. For the newly grown wood that had been treated with 10 mmol/L and 100 mmol/L TEOS, the microfibril angle increased by 2.4° and 5.5°, respectively compared with that of the wood from the previous year; meanwhile, it was 3.0° and 8.2° higher than that of Group CK, i.e., the untreated group, indicating that the changes in the microfibril angle were related to the concentration of the treatment. In Group S—namely, the group that had been treated with SiO_2_—the microfibril angle of the newly grown wood had an increase of 4.8° and 4.2° compared with that of the wood from the previous year and that of the Group CK, respectively. Since the Wood Microfibril Angle (MFA) had an effect on the wood shrinkage, swelling anisotropy and wood mechanical properties [24], based on the increasing trend of MFA shown in this study, it was assumed that the compression strength parallel to a grain of treated wood would be decreased, whereas the longitudinal shrinkage and swelling would go up. On the other hand, the presence of silicon might work as a compensation for the degradation or deterioration of this property.

### 3.7. Experiment Analysis of the Flakes Tensile and Wood Basic Density

After treatment with different sustained-release drip conditions, the basic density of the wood and the elastic modulus and tensile strength of the wood flakes were recorded and are shown in Table 3. It can be clearly seen that the density of all the treated wood has obviously increased. The density of the new wood treated with 10 mmol/L TEOS is the highest, and the elastic modulus and tensile strength have increased. Other treatments show that the elastic modulus and tensile strength of the wood have decreased to different degrees. Especially, the tensile strength of the fresh wood treated with 100 mmol/L TEOS is seriously reduced; its modulus of elasticity is too small and the data has not been displayed on the micro-tensile mechanical experimental instrument after many measurements. It may be that the tree happens to grow a knot at the location of the injection point that seriously affects the mechanical properties of the wood and causes the T3 data to be abnormal. The T1 and S treatment groups had a reduced modulus of elasticity and tensile strength compared to the control group. One reason for this is that the inorganic matter generated by the reaction of TEOS occupies the crystalline zone of the cellulose, reducing the crystallinity of the wood and lowering the modulus of elasticity. On the other hand, the inorganic matter generated is not chemically bonded well to the wood, and the inorganic matter is not uniformly distributed, which reduces the mechanical strength of the composite. This is inconsistent with the findings of Devi, R. et al. [25], who found that adding nano-TiO_2_ gel to wood can improve its tensile properties. Compared with the control group, the modulus of elasticity and tensile strength of the T_2_ treatment group increased.

It may be that the TEOS concentration of 10 mmol/L is the most suitable for the moisture reaction in wood, thereby promoting the reaction and producing a good chemical bonding with the wood. The mechanical properties of the wood are thereby enhanced. Furthermore, the measured change in the microfilament inclination angle showed the smallest increase in the microfilament inclination angle in the new wood treated with 10 mmol/L TEOS compared to the other treatment groups, while the microfilament inclination angle was negatively correlated with the mechanical strength of the wood [26], resulting in the greatest modulus of elasticity and tensile strength in the T2 treatment group.

## 4. Conclusions

With TEOS and silica sol as precursors, this experiment obtained wood/inorganic composites via the self-organization of wood by the standing tree slow-release drip technique. Light microscopy demonstrated that the inorganic substances were yellowish brown and widely existed in vessels, wood fibers and ray cells. For the newly formed wood, such impregnation treatment contributed to a decrease in the diameter and tissue ratio of the vessels and an increase in the fiber–tissue ratio, double wall thickness and microfibril angle. Moreover, such changes were related to the concentration of precursors. EDS analysis proved that the sediments in the wood cells were an elemental composition of C, O, Si, K and Ca. It was further confirmed, by XPS, that the newly 是wood contained silicon oxide, and the inorganic substance presented different contents in the newly formed wood, injection point and previously formed wood. The FTIR test demonstrated that no chemical reactions, but rather physical attachment or depositions, occurred between the wood and the inorganic substances. After treatment with the appropriate concentration, the tensile strength and modulus of elasticity parallel to the grain of wood underwent an increase.

## Figures and Tables

**Table 1:** Vessel diameter, vessel proportion, fiber proportion and fiber double wall thickness in different positions of poplar under different treatment conditions. The values after the +/− sign represent the standard deviation: T1, T2 and T3 for the 110,100 mmol/L TEOS modification treatment group, S for the SiO_2_ colloidal injection group, CK for the unmodified treatment group, PW for the previously grown wood, IP for the injection point and NW for the newly grown wood.

Treating Conditions	Locations	Vessel Diameter/μm	Vessel Proportion/%	Fiber Double Wall Thickness/μm	Fiber Propotion/%
CK	NW	162.33 ± 28.36	36.39 ± 3.45	6.32 ± 2.65	58.97 ± 3.65
S	NW	101.44 ± 16.25	17.15 ± 3.26	7.58 ± 1.07	72.72 ± 2.56
	IP	99.17 ± 17.92	31.43 ± 2.59	6.35 ± 1.00	60.18 ± 2.39
	PW	190.03 ± 18.24	31.51 ± 3.45	5.42 ± 0.76	61.16 ± 2.57
T1	NW	118.83 ± 10.29	21.31 ± 4.05	6.97 ± 1.09	70.82 ± 3.26
	IP	122.27 ± 11.26	26.88 ± 5.03	4.82 ± 0.58	66.00 ± 3.16
	PW	157.67 ± 13.25	40.45 ± 2.69	3.68 ± 0.62	52.73 ± 2.19
T2	NW	133.41 ± 14.26	17.06 ± 3.56	6.61 ± 1.12	76.74 ± 1.85
	IP	88.24 ± 13.87	21.12 ± 4.61	6.14 ± 0.97	70.90 ± 2.30
	PW	190.81 ± 12.29	34.85 ± 2.35	4.82 ± 0.66	59.54 ± 1.29
T3	NW	101.30 ± 20.56	32.98 ± 5.64	6.42 ± 0.80	61.48 ± 2.19
	IP	80.04 ± 32.26	28.80 ± 2.36	5.98 ± 1.32	65.50 ± 3.46
	PW	154.55 ± 29.68	40.82 ± 1.98	5.37 ± 0.55	53.45 ± 2.32

**Table 2:** Microfibril angle in different positions of poplar under different treatment conditions. The values after the +/− sign represent the standard deviation; PW represents the previously grown wood; IP represents the injection point; NW represents the newly grown wood; T1, T2, T3 represent the 110,100 mmol/L TEOS modification treatment group; S represents the SiO_2_ colloidal injection group; CK represents the unmodified treatment group.

Treatment Conditions	T1		T2		T3		S		CK
Measuring position	PW	NW	PW	NW	PW	NW	PW	NW	
Microfibril angle/°	26.7 ± 2.9	29.1 ± 3.5	23 ± 2.9	25.4 ± 3.7	25.1 ± 3.0	30.6 ± 2.4	22.8 ± 4.0	27.6 ± 4.1	22.4 ± 2.5

**Table 3:** Tensile properties of the poplar flakes treated and wood basic density by different sustained-release drip conditions, The values after the +/− sign represent the standard deviation; T1, T2, T3 represent the 110,100 mmol/L TEOS modification treatment group; S represents the SiO_2_ colloidal injection group; CK represents the unmodified treatment group.

Treatment Conditions	T1	T2	T3	S	CK
Modulus of elasticity/GPa	3.323 ± 0.015	5.372 ± 0.014	—	4.047 ± 0.016	4.989 ± 0.015
Tensile Strength/MPa	80.124 ± 6.14	130.586 ± 4.36	14.927 ± 6.05	105.235 ± 4.46	111.762 ± 5.51
wood basic density/g×cm^−3^	0.429 ± 0.04	0.452 ± 0.03	0.409 ± 0.03	0.465 ± 0.03	0.387 ± 0.04

## Data Availability

The data presented in this study are available on request from the corresponding author.
